# New distribution records of two bamboo species in Yunnan, China with description of the inflorescence for *Melocalamus yunnanensis* (Poaceae, Bambusoideae)

**DOI:** 10.3897/phytokeys.62.7276

**Published:** 2016-03-25

**Authors:** Yu-Xiao Zhang, Xia-Ying Ye, Hong-Mei Yang, Xian-Zhi Zhang, Ping-Yuan Wang, De-Zhu Li

**Affiliations:** 1 Germplasm Bank of Wild Species, Kunming Institute of Botany, Chinese Academy of Sciences, Kunming 650201, China; 2 Key Laboratory for Plant Diversity and Biogeography of East Asia, Kunming Institute of Botany, Chinese Academy of Sciences, Kunming 650201, China; 3 Kunming College of Life Science, University of Chinese Academy of Sciences, Kunming 650201, China; 4 Key Laboratory of Southern Subtropical Plant Diversity, Fairylake Botanical Garden, Shenzhen & Chinese Academy of Sciences, Shenzhen 518004, China; 5 College of Forestry, Northwest A & F University, Yangling, Shaanxi 712100, China; 6 Xishuangbanna Tropical Botanical Garden, Chinese Academy of Sciences, Menglun, Yunnan 666303, China

**Keywords:** *Ampelocalamus actinotrichus*, *Neomicrocalamus prainii*, biogeographic implications, molecular discrimination, inflorescence

## Abstract

*Ampelocalamus actinotrichus* (Merrill & Chun) S. L. Chen, T. H. Wen & G. Y. Sheng and *Neomicrocalamus prainii* (Gamble) P. C. Keng are reported with new distribution records in southern and southeastern Yunnan, China, respectively. *Ampelocalamus actinotrichus* was previously recorded to be endemic to Hainan, China, and *Neomicrocalamus prainii* to be distributed in southern Tibet and western Yunnan in China, northeastern India, and Burma. The identities of individuals collected in southern and southeastern Yunnan of these two species are confirmed by molecular evidence. The new distribution record of *Ampelocalamus actinotrichus* provides a case at the species level for confirming floristic affinities of southern Yunnan and Hainan Island in south China. The disjunct distribution of *Neomicrocalamus prainii* in Yunnan is concordant with the ecogeographical diagonal line from northwestern Yunnan to southeastern Yunnan and this may imply a tropical origin of this species. In addition, the inflorescence of *Melocalamus yunnanensis* (T. H. Wen) T. P. Yi is described.

## Introduction

Although morphological descriptions, distributions, and pictures of most bamboo species and genera in China have been recorded in the *Flora Reipublicae Popularis Sinicae*
(FRPS) (Keng and Wang 1996), the *Flora of China* (Li et al. 2006), and the *Iconographia Bambusoidearum Sinicarum* (Yi et al. 2008), there are still many aspects that need to be changed incorporating findings from molecular phylogenetic analyses, the re-evaluation of morphological characters, and additional field investigations. During our field work from 2012 to 2014, two distinct bamboo species were noted. One occurs in Xishuangbanna, southern Yunnan, China, which was initially identified as “*Ampelocalamus menglaensis*” Hsueh & F. Du *nom. nud.* (Du et al. 1999), but closely resembled *Ampelocalamus actinotrichus* (Merrill & Chun) S. L. Chen, T. H. Wen & G. Y. Sheng. The other is distributed in Malipo, southeastern Yunnan, China, where it is called “*teng zhu*” (climbing bamboo) by local people, and was tentatively identified as *Neomicrocalamus prainii* (Gamble) P. C. Keng.

*Ampelocalamus actinotrichus*, the type species of the genus *Ampelocalamus* S. L. Chen, T. H. Wen & G. Y. Sheng (Chen et al. 1981), belongs to the tribe Arundinarieae (BPG 2012), and is endemic to the island of Hainan, China, according to the FRPS and the *Flora of China*. *Neomicrocalamus prainii* is the type species of the genus *Neomicrocalamus* P. C. Keng which belongs to the subtribe Bambusinae (Bambuseae) (Keng 1983, BPG 2012), and is distributed in southern Tibet and western Yunnan of China (Keng and Wang 1996, Li et al. 2006), and northeastern India (Meghalaya and Nagaland) (Ohrnberger 1999, Kumari and Singh 2013). If the two species discovered in southern and southeastern Yunnan, are indeed *Ampelocalamus actinotrichus* and *Neomicrocalamus prainii*, respectively, their collections would represent new distribution records in China for those species.

In order to further confirm the identities of these two species in southern and southeastern Yunnan, molecular phylogenetic analyses were carried out. Previous molecular phylogenetic studies demonstrated that plastid regions had low resolution at species and generic levels in Arundinarieae (Triplett and Clark 2010, Zeng et al. 2010), and conspicuous conflicts between plastid and nuclear phylogenetic tree topologies have been found (Zhang et al. 2012, Yang et al. 2013). Moreover, nuclear phylogenies were more congruent with morphology-based classifications and had higher resolutions than plastid phylogenies. Therefore, we selected one nuclear gene *LEAFY* (Yang et al. 2013) for the molecular identification of the “*Ampelocalamus menglaensis*” sample. The standard DNA barcoding plastid regions had been found to possess a certain discriminative power at species and generic levels in the subtribe Bambusinae and can be obtained by direct sequencing, while obtaining nuclear genes may involve cloning which requires more time and resources (Yang et al. 2010, Goh et al. 2013). Given their convenience and efficiency, we selected four plastid regions (*rbc*L–*psa*I, *rpl*32–*trn*L, *trn*G–*trn*T(g), *trn*G–*trn*T(t)) that show higher levels of variation than standard DNA barcoding markers (Zhang et al. 2013), for identifying the “*teng zhu*” sample.

In addition to the two distinct bamboo species, we collected flowering specimens of *Melocalamus yunnanensis* (T. H. Wen) T. P. Yi in the field and the Bamboo Garden of Xishuangbanna Tropical Botanical Garden. The inflorescence of this species is described in this paper, and its identity as a member of the genus *Melocalamus* Bentham reconfirmed.

## Materials and methods

### Field collections

Specimens for morphological observations and silica gel-dried leaf samples of “*Ampelocalamus menglaensis*” and *Melocalamus yunnanensis* were collected in August 2012 and April 2013 in southern Yunnan (Mengla, Xishuangbanna) and of “*teng zhu*” in October 2013 and August 2014 in southeastern Yunnan. The distribution map was created using DIVA-GIS (http://www.diva-gis.org).

### Morphological observations

The specimens collected in southern Yunnan were compared with specimens of *Ampelocalamus actinotrichus* from Hainan Island. The specimens of “*teng zhu*” from southeastern Yunnan were compared with those of *Neomicrocalamus prainii* from northwestern Yunnan and southern Tibet.

The inflorescence of *Melocalamus yunnanensis* from the Bamboo Garden of Xishuangbanna Tropical Botanical Garden was compared with specimens of this species from Jiangcheng, southern Yunnan. The glumes, lemma, palea, ovary, style, and stamens were observed under a hand-lens (30×).

### Taxon sampling

The phylogeny of Arundinarieae based on *LEAFY* indicated that the genus *Ampelocalamus* is monophyletic, except for *Ampelocalamus calcareus* C. D. Chu & C. S. Chao, and sister to *Drepanostachyum* P. C. Keng and *Himalayacalamus* P. C. Keng (Yang et al. 2013). Thus, samples of the latter two genera were used as outgroup in our study. We aimed to elucidate the identity of “*Ampelocalamus menglaensis*”, not to infer phylogenetic relationships. Therefore, only those taxa closely related to *Ampelocalamus actinotrichus* were included (Table 1).

**Table 1.** Plant materials, voucher information, and GenBank accession numbers of the samples used in the phylogenetic analyses.

Taxon	Voucher	Locality	GenBank accession number				
			Leafy	*rbc*L-*psa*I	*rpl32-trn*L	*trn*G-*trn*T(g)	*trn*G-*trn*T(t)
**Arundinarieae Nees ex Asch. & Graebn.**							
*Ampelocalamus actinotrichus* (Merr. and Chun) S.L. Chen *et al*.	MPF10001	Ledong, Hainan, China	KM264728	—	—	—	—
*Ampelocalamus actinotrichus* (Merr. and Chun) S.L. Chen *et al*.	MPF10003	Ledong, Hainan, China	KM264729 KM264730	—	—	—	—
*Ampelocalamus actinotrichus* (Merr. and Chun) S.L. Chen *et al*.	12167	Mengla, Yunnan, China	KR057489	—	—	—	—
*Ampelocalamus actinotrichus* (Merr. and Chun) S.L. Chen *et al*.	13007	Mengla, Yunnan, China	KR057490	—	—	—	—
*Ampelocalamus actinotrichus* (Merr. and Chun) S.L. Chen *et al*.	13012	Mengla, Yunnan, China	KR057491	—	—	—	—
*Ampelocalamus luodianensis* T.P. Yi & R.S. Wang	MPF10052	Luodian, Guizhou, China	KM264732 KM264733	—	—	—	—
*Ampelocalamus melicoideus* (P.C. Keng) D.Z. Li & Stapleton	MPF10142	Nanchuan, Chongqing, China	KM264735	—	—	—	—
*Ampelocalamus microphyllus* (Hsueh & T.P. Yi) Hsueh & T. P. Yi	MPF10123	Wuxi, Chongqing, China	KM264734	—	—	—	—
*Ampelocalamus patellaris* (Gamble) Stapleton	Zhang07075	Lvchun, Yunnan, China	KM264785	—	—	—	—
*Drepanostachyum ampullare* (T.P. Yi) Demoly	GLM081860	Shannan, Xizang, China	KM264793	—	—	—	—
*Himalayacalamus falconeri* (Munro) P.C. Keng	GLM081524	Jilong, Xizang, China	KM264791 KM264792	—	—	—	—
**Bambuseae Kunth ex Dumort.**							
*Bambusa lapidea* McClure	12152	Jiangcheng, Yunnan, China	—	KF764854	KF764906	KF765049	KF765025
*Bambusa teres* Buchanan-Hamilton ex Munro	12204	Yingjiang, Yunnan, China	—	KF764853	KF764907	KF765050	KF765026
*Bonia amplexicaulis* (L. C. Chia et al.) N. H. Xia	12329	Pingxiang, Guangxi, China	—	KF764851	KF764908	KF765051	KF765027
Bonia saxatilis (L. C. Chia et al.) N. H. Xia var. saxatilis	12327	Bama, Guangxi, China	—	KF764852	KF764909	KF765052	KF765028
*Dendrocalamus barbatus* Hsueh & D. Z. Li	12151	Jiangcheng, Yunnan, China	—	KF764849	KF764912	KF765055	KF765021
*Dendrocalamus brandisii* (Munro) Kurz	12142	Jiangcheng, Yunnan, China	—	KF764848	KF764914	KR057481	KF765023
*Melocalamus yunnanensis* (T. H. Wen) T. P. Yi	12153	Jiangcheng, Yunnan, China	—	KR057474	KR057467	KR057482	KR057461
*Neomicrocalamus prainii* (Gamble) P.C. Keng	LL07236	Motuo, Xizang, China	—	KR057476	KR057469	KR057484	KR057463
*Neomicrocalamus prainii* (Gamble) P.C. Keng	LL07567	Linzhi, Xizang, China	—	KR057477	KR057470	KR057485	KR057464
*Neomicrocalamus prainii* (Gamble) P.C. Keng	ZXZ11027	Gongshan, Yunnan, China	—	KR057480	KR057473	KR057488	—
*Neomicrocalamus prainii* (Gamble) P.C. Keng	13045	Malipo, Yunnan, China	—	KR057475	KR057468	KR057483	KR057462
*Neomicrocalamus prainii* (Gamble) P.C. Keng	YXY150	Malipo, Yunnan, China	—	KR057478	KR057471	KR057486	KR057465
*Neomicrocalamus prainii* (Gamble) P.C. Keng	YXY151	Xichou, Yunnan, China	—	KR057479	KR057472	KR057487	KR057466

In order to confirm the identity of “*teng zhu*”, representatives of the subtribe Bambusinae were chosen according to Yang et al. (2008), including taxa of *Bambusa* Schreber, *Bonia* Balansa, *Dendrocalamus* Nees, and *Melocalamus* Bentham (Table 1).

Voucher specimens of all samples are deposited at the herbarium of the Kunming Institute of Botany, Chinese Academy of Sciences (KUN).

### DNA isolation, amplification, and sequencing

Total genomic DNA was extracted from silica gel-dried leaves using a modified CTAB procedure (Doyle and Doyle 1987). Primers and protocols for the PCR amplification of the nuclear gene *LEAFY* and four plastid regions (*rbc*L–*psa*I, *rpl*32–*trn*L, *trn*G–*trn*T(g), *trn*G–*trn*T(t)) followed prior studies (Yang et al. 2013, Zhang et al. 2013). PCR products were checked on 1% agarose gels, and purified using ExoSAP–IT (USB, Cleveland, OH, USA). Double–stranded and purified PCR products were sequenced by the dideoxy chain termination method with ABI PRISM Bigdye Terminator Cycle Sequencing Ready Reaction Kit with AmpliTaq DNA polymerase FS (Perkin Elmer, Waltham, MA, USA). PCR reactions and programs were chosen according to the recommendations of the handbook, with slight modifications in some cases. Bidirectional sequencing was performed on an ABI 3730xl automated sequencer.

### Phylogenetic analyses

Sequences were assembled and edited with SeqMan (DNA STAR package; DNA Star Inc., Madison, WI, USA), aligned by MUSCLE (Edgar 2004), and adjusted manually where necessary. Informative indels introduced by the sequence alignment were coded as binary characters using the simple indel coding method of Simmons and Ochoterena (2000). Due to no obvious conflicts among individual plastid trees, we used a combined data set of four plastid regions for phylogenetic analyses. All data matrices are available on request from the first author. Sequences newly obtained in this study have been deposited in GenBank (Table 1). Accession numbers initialed with ‘KM’ and ‘KF’ were downloaded directly from GenBank, and the others were obtained in this study.

For phylogeny reconstructions, we used three methods, namely maximum parsimony (MP), maximum likelihood (ML), and Bayesian inference (BI). The MP and ML analyses were conducted with PAUP* version 4.0b10 (Swofford 2002). For the ML analyses, the best-fitting models were selected using jModeltest v2.1.4 under the Akaike Information Criterion (AIC) (Darriba et al. 2012). The TIM3 model and TPM1uf+G model were selected for *LEAFY* and combined plastid regions, respectively. MP and ML analyses were conducted with the same parameter setting for the heuristic search and the bootstrap calculation. The heuristic search was performed with 1000 random addition sequence replicates and TBR branch swapping, MULTREES option in effect. Strict consensus trees were calculated for all MP analyses. Branch support was estimated with 1000 bootstrap replicates (Felsenstein 1985) using the heuristic search method as described above (with 100 random addition sequence replicates). The same models were used for Bayesian analyses with MrBayes version 3.2.5 (Ronquist et al. 2012). Two independent runs were conducted simultaneously starting with random trees, and each run consisted of one cold and three hot chains. Chains were run for 150000 generations for *LEAFY* and 200000 generations for the combined plastid data set, and trees sampled every 100 generations. The average standard deviation of split frequencies between both runs reached a value below 0.01. The convergence of the chains and the number of trees to be discarded were determined using Tracer version 1.6 (http://tree.bio.ed.ac.uk/software/tracer). The initial 25% trees were discarded as burn-in, and the remaining trees were used to construct a 50% majority-rule consensus trees. MP and ML bootstrap values ≥ 50% and BI posterior probabilities ≥ 0.95 were labeled on the tree branches.

## Results

### Phylogenetic analysis

The aligned length of *LEAFY* was 738 bp, and six indels were coded as additional absent/present (0/1) characters, giving a total of 744 characters in the MP matrix, of which 26 were parsimony-informative. Sequences of the four plastid regions were obtained for all samples, except *trn*G-*trn*T(t) for the sample ZXZ11027. The combined plastid matrix was 3737 bp long and included 14 indel characters in the MP matrix, 113 of which were parsimony-informative. There were inversions in the *rbc*L–*psa*I and *rpl*32–*trn*L sequences (Zeng et al. 2010, Zhang et al. 2013) and gaps were introduced to separate the inverted regions to avoid overweighting the inversions. Those gaps were not scored and treated as missing data.

**Figure 1.**
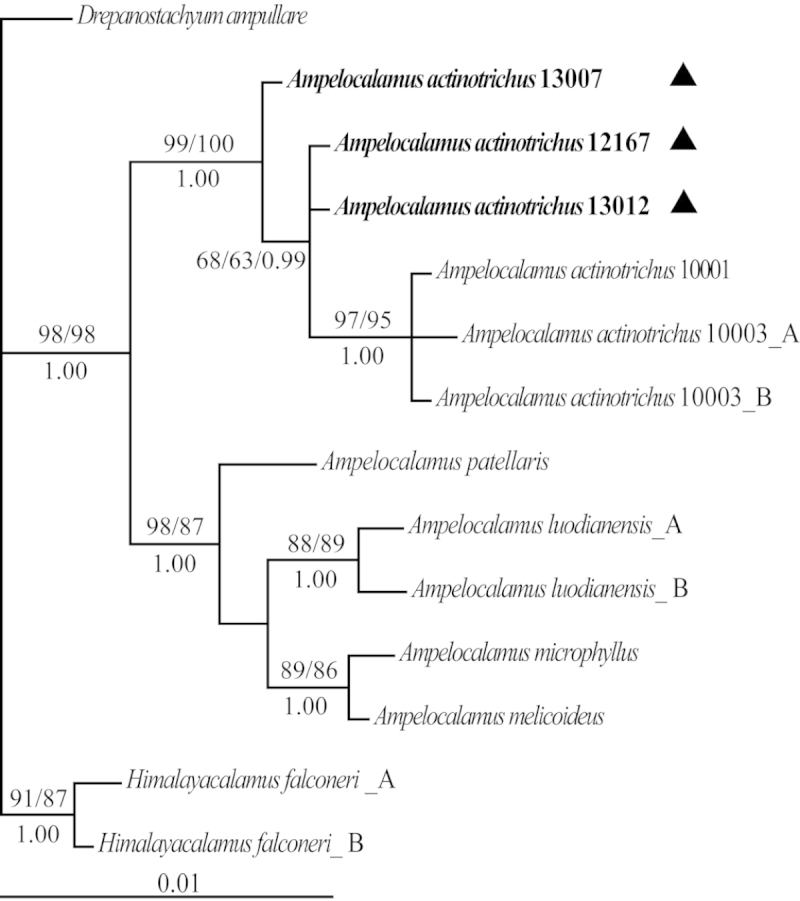
Phylogram of the 50% majority-rule consensus tree from Bayesian analysis of *LEAFY* sequences. Bootstrap values over 50% and Bayesian posterior probabilities over 0.95 are shown along branches (MP/ML/BI). Individuals of *Ampelocalamus actinotrichus* from Yunnan are in bold and indicated by solid triangles. Letters **A** and **B** after taxon names denote different alleles of *LEAFY*.

The 50% majority-rule consensus tree from BI for *LEAFY* is presented in Fig. 1. The ML tree and the MP strict consensus tree were consistent with the Bayesian tree except for a few branches with very low support (not shown). The statistical support was shown along the branches (MP/ML/BI). All individuals of *Ampelocalamus* formed a clade with high support (MP/ML/BI = 98/98/1.00). The samples of “*Ampelocalamus menglaensis*” (voucher numbers 12167, 13007, and 13012) fell with the *Ampelocalamus actinotrichus* samples collected from Hainan.

**Figure 2.**
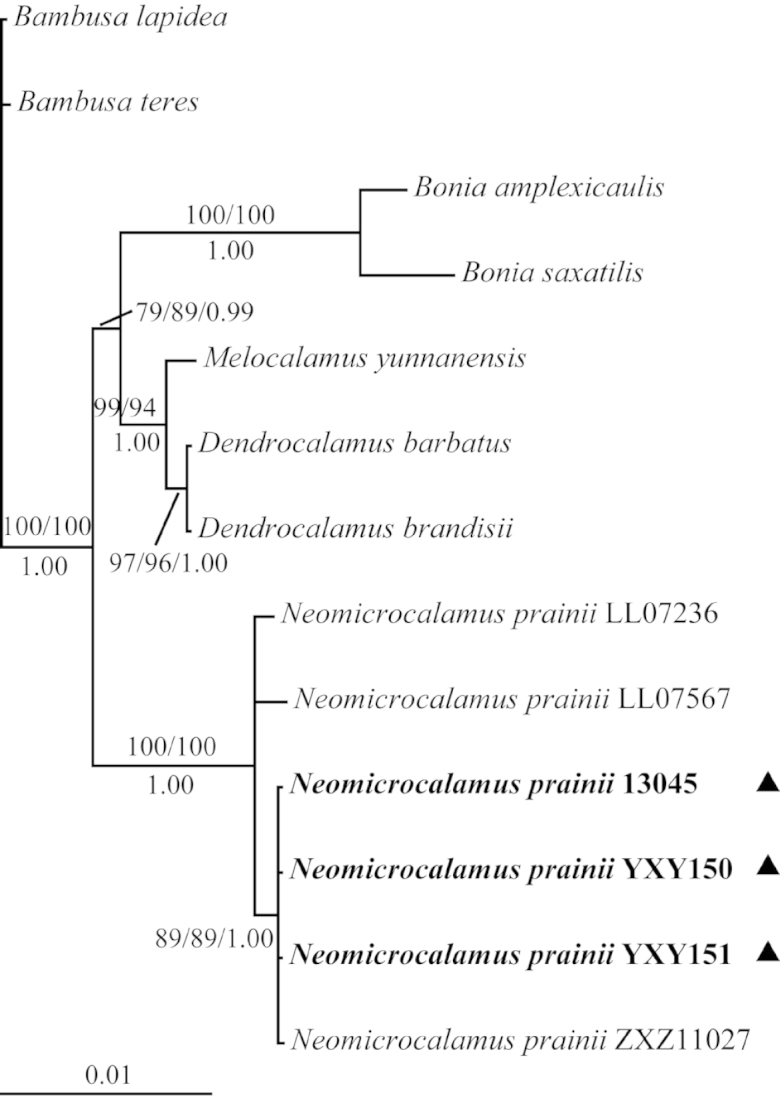
Phylogram of the 50% majority-rule consensus tree from Bayesian analysis of the combined plastid data set (*rbc*L–*psa*I, *rpl*32–*trn*L, *trn*G–*trn*T(g), and *trn*G–*trn*T(t)). Bootstrap values over 50% and Bayesian posterior probabilities over 0.95 are shown along branches (MP/ML/BI). Individuals of *Neomicrocalamus prainii* from southeastern Yunnan are in bold and indicated by solid triangles.

The 50% majority-rule consensus tree from BI for the combined plastid data set is presented in Fig. 2. The ML tree and the MP strict consensus tree were the same with the Bayesian tree. The statistical support is shown along the branches (MP/ML/BI). Individuals of “*teng zhu*” collected from southeastern Yunnan and *Neomicrocalamus prainii* ZXZ11027 from northwestern Yunnan fell in one clade with high support (MP/ML/BI = 89/89/1.00), and they subsequently formed a monophyletic group with *Neomicrocalamus prainii* from Tibet (MP/ML/BI = 100/100/1.00).

### Description of the inflorescence of *Melocalamus yunnanensis*

#### Melocalamus yunnanensis

Taxon classificationPlantaePoalesPoaceae

(T. H. Wen) T. P. Yi, 2007

Fig. 3

Racemobambos yunnanensis T. H. Wen, J. Bamboo Res. 5: 11. 1986; Neomicrocalamus yunnanensis (T. H. Wen) Ohrnberger, The Bamboos of the World: Introduction to the Work, 4: 19.1997.
Melocalamus yunnanensis (T. H. Wen) T. P. Yi, J. Sichuan Forest. Sci. Tech. 28: 18. 2007.

##### Type.

CHINA. Yunnan: Jinping, *W. W. Zhou ZP. 83311* (holotype, ZJFI).

Culms scrambling, 6–15 m, 5–10 mm in diam.; internodes 20–60 cm, smaller culms solid or nearly so, white pubescent, especially dense below corky nodes. Dominant branches equal in size to culm, other branches slender and many. Culm leaf sheaths brown scabrous, lower portion very tough, upper papery and thin, margins glabrous, shoulders protruding conspicuously; ligules inconspicuous; auricles and oral setae absent; blades lanceolate, recurved. Leaves 3-4 per ultimate branch; leaf sheaths slightly pubescent, margins ciliate; ligules 1–1.5 mm; auricles absent; oral setae erect to spreading, short; blades lanceolate, 4–7 × 0.8–1.3 cm, glabrous, veins 4 pairs, without tessellation. Flowering branches with or without leaves, internodes with dense white pubescence. Pseudospikelets 0.8-1.2 cm, several to many clustered on nodes, glomerate, 2–3 florets for each pseudospikelet with the top one sterile; rachilla ca. 1.5 mm; glumes 2, 2–4 mm; lemma purple red, 5–6 mm, glabrous; palea a little longer than lemma, purple red, 6–7 mm, 2–keeled, glabrous; lodicules 3, nearly equal, margins ciliate; stamens 6, yellow; ovary ovate–lanceolate; style 1, stigmas 2, plumose. Caryopsis unknown.

##### Voucher specimens.

**CHINA. Yunnan**: Jiangcheng, 860 m, 22°28.429'N, 101°29.938'E, 12 August 2012, *Y. X. Zhang, Y. X. Xu & M. Y. Zhou 12153, 12154* (KUN); Mengla, 790 m, 21°36.55’N, 101°33.683’E, 8 April 2013, *Y. X. Zhang &Y. X. Xu 13006*, *13009* (KUN); Menglun, the Bamboo Garden of Xishuangbanna Tropical Botanical Garden (cultivated), 12 March 2012, *P. Y. Wang C130104* (HITBC).

**Figure 3.**
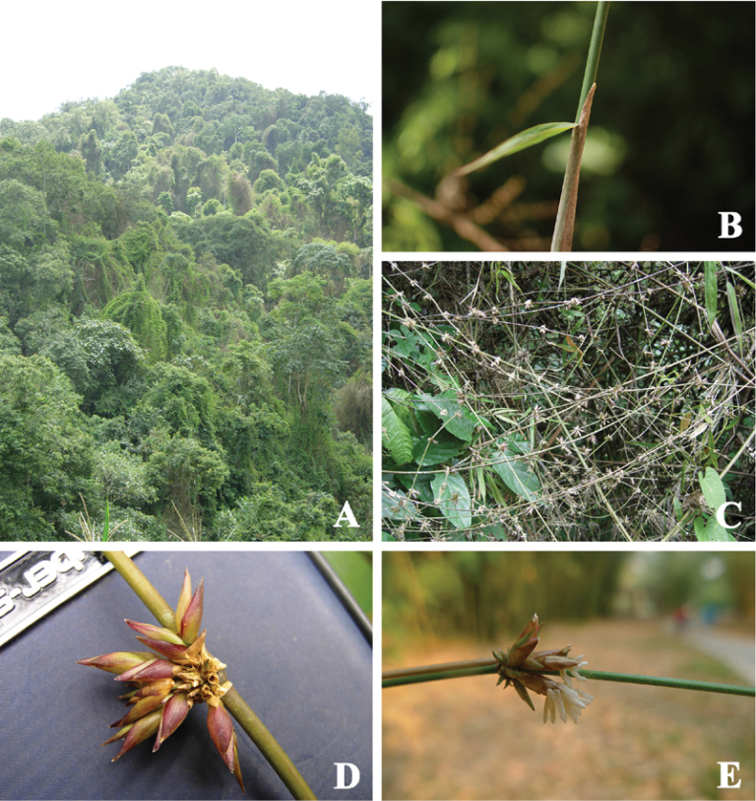
*Melocalamus yunnanensis*. **A** Habitat (*12153*) **B** Culm leaf (*12153*) **C** Flowering branches (*12154*) **D–E** Pseudospikelets (**D** *12154*, **E** *C130104*).

## Discussion

### *Ampelocalamus actinotrichus* in southern Yunnan and its biogeographic implications

**Figure 4.**
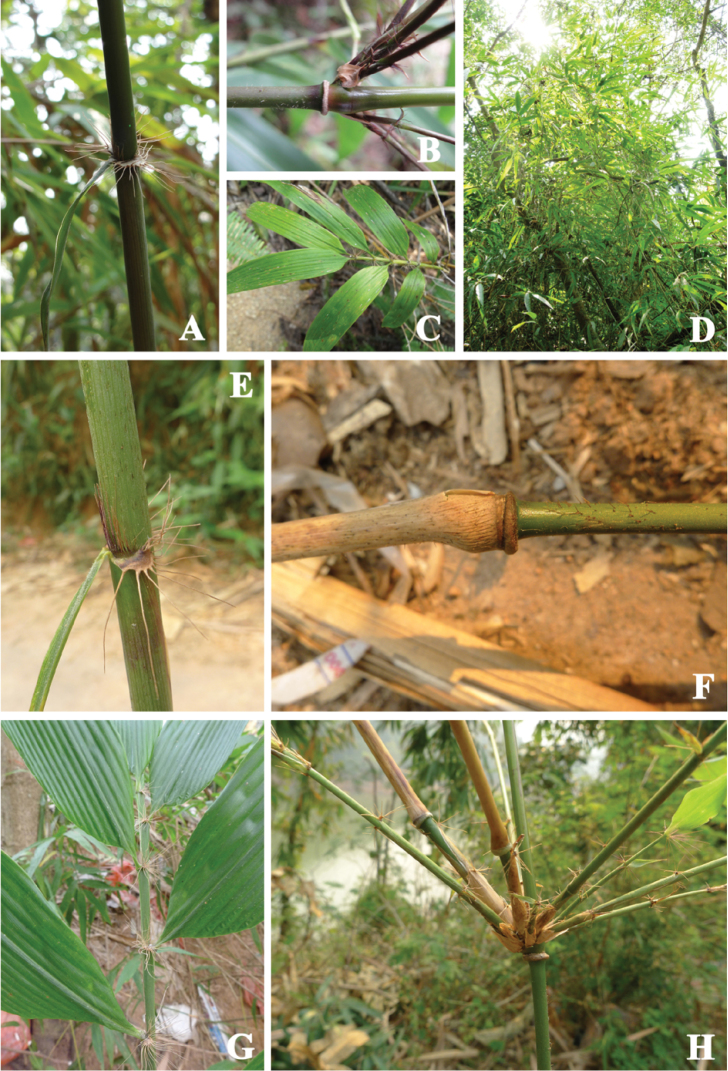
*Ampelocalamus actinotrichus* from Hainan and Yunnan, China. **A–C** Individuals from Hainan **A** Culm leaf (*MPF10001*) **B** Branches and young culm (*MPF10001*) **C** Leaves (*MPF10003*) **D–H** Individuals from Yunnan **D** Culms climbing on trees (*13012*) **E** Culm leaf (*13001*, collected at the same locality with *12167*) **F** Young culm (*13001*) **G** Leaves (*13001*) **H** Branches (*13001*).

Our collections (specimens 12167, 13007, and 13012) were identified as *Ampelocalamus menglaensis* *nom. nud.* by bamboo taxonomists. This species was initially published without proper description and designation of the type specimen, and it was reported that this bamboo was originally distributed in Mengla, Yunnan, China (Du et al. 1999). In the *Flora Yunnanica* (Sun et al. 2003), it was suggested that this species could represent a misidentification of *Dinochloa puberula* McClure and was not included in the genus *Ampelocalamus*. Later, Wang (2004) described this species briefly in Chinese with two colored photos in his book *Ornamental Bamboos*. Based on the brief description and our observations in the field, we inferred that this species should be *Ampelocalamus actinotrichus*. Major morphological diagnostic characters included: culms pendulous or scrambling; rhizomes pachymorph; internodes with sparsely brown setae initially; nodal sheath scars prominent; branches two to several, occasionally with one dominant branch replacing the main culm and scrambling to other plants; culm leaves tardily deciduous, much shorter than internodes, sheaths with sparse setae abaxially, auricles ovate, oral setae radiate and initially purple, margins of ligules with long and purple hairs, blades lanceolate; leaf sheaths glabrous or with sparse setae abaxially, auricles kidney-shaped, oral setae radiate and initially purple, ligules fimbriate, blades with long pubescence on both epidermises (Fig. 4).

Yang (2005) analyzed the anatomy of leaves and roots of 13 species of *Drepanostachyum* P.C. Keng and *Ampelocalamus*, including *Ampelocalamus actinotrichus* and *Ampelocalamus menglaensis* *nom. nud.* *Ampelocalamus actinotrichus* and *Ampelocalamus menglaensis* *nom. nud* had similar features but distinct from other species, such as two layers of mesophyll cells in leaf transverse sections, large fusoid cells, fewer prickles on abaxial and adaxial epidermises, microhairs developed. Moreover, our molecular phylogenetic analyses demonstrated that our collections from Yunnan grouped together with *Ampelocalamus actinotrichus* from Hainan, and there was a little genetic divergence between these two biogeographic entities (Fig. 1). On grounds of the aforementioned evidence we can confirm that *Ampelocalamus menglaensis* *nom. nud.* is conspecific with *Ampelocalamus actinotrichus*.

**Figure 5.**
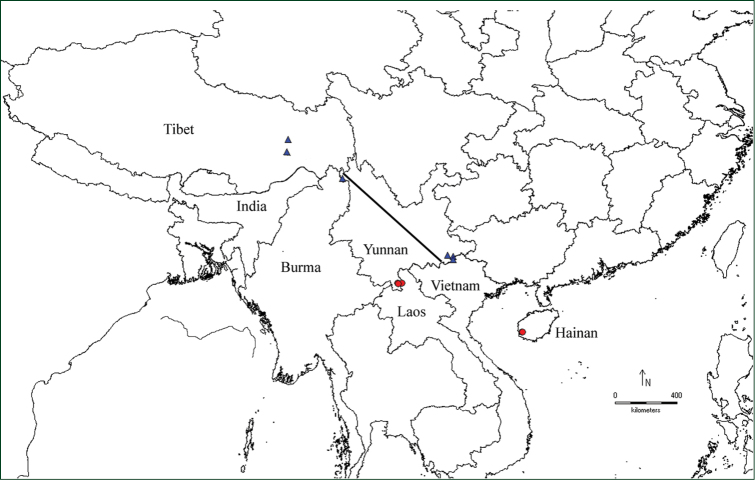
Distribution of *Ampelocalamus actinotrichus* and *Neomicrocalamus prainii* in China (based on specimens cited in this paper). Solid triangles: *Neomicrocalamus prainii*; solid circles: *Ampelocalamus actinotrichus*; the solid line: the ecogeographical diagonal line from northwestern Yunnan to southeastern Yunnan (Li 1994).

*Ampelocalamus actinotrichus* was recorded to occur only on the island of Hainan before our confirmation of its occurence in southern Yunnan (Fig. 5). During our field investigations in Mengla, Xishuangbanna, southern Yunnan we went to the border of China and Laos (collecting voucher specimens 13012 and 13013; 13013 was not used here for the molecular analyses). We were told by the local people that this bamboo could also be found in northern Laos. Chen and Wen (2008) reported that this bamboo is distributed in tropical montane rainforests of Mengsong, southern Yunnan, which is adjacent to Burma. It suggests that *Ampelocalamus actinotrichus* may be distributed in Burma. Additional field explorations are needed to clarify whether this species is more widely distributed on the Indo-China peninsula. The new distribution record of this species has a great significance in resolving the origin and divergence of the genus *Ampelocalamus*.

Mengla and Mengsong are both parts of Xishuangbanna, in southern Yunnan. The flora of Xishuangbanna is part of the Indo-Malesian flora, and has a close affinity with floras of adjacent areas (i.e., southern China including tropical Guangxi and Hainan, Burma, Laos, Thailand, and Vietnam) (Zhu 1994, Zhu et al. 2001, Zhu and Roos 2004). The distribution of *Ampelocalamus actinotrichus* in both Hainan and southern Yunnan (and likely in Burma and Laos) provides a case at the species level for confirming the affinity of floras across southern China including Xishuangbanna (and likely adjacent areas).

### *Neomicrocalamus prainii* in southeastern Yunnan and its biogeographic implications

**Figure 6.**
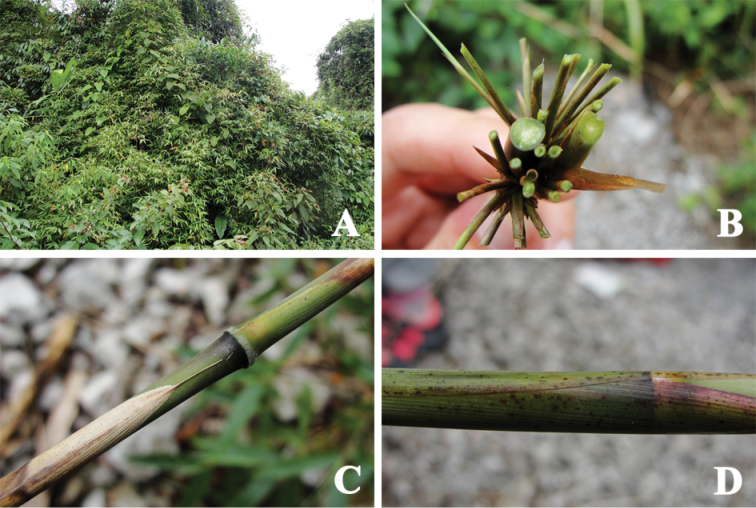
*Neomicrocalamus prainii* (*YXY150*). **A** Habitat **B** Branches **C–D** Culm leaves.

From the point of view of morphology, individuals of “*teng zhu*” (specimens 13045, YXY150, YXY151) from southeastern Yunnan share many features of *Neomicrocalamus prainii*, including culms scrambling, nearly solid, branches many with the dominant branch equal in size to the culm and other small branches seldom branching again, culm leaf sheaths with purple–brown spots and white pubescence abaxially, culm leaf blades acicular, and others (Fig. 6). The habitats of “*teng zhu*” in southeastern Yunnan are mainly rocky mountains which are similar to those we found in Gongshan, northwestern Yunnan (voucher No. ZXZ11027). The molecular phylogenetic analyses illustrated that those individuals fell with *Neomicrocalamus prainii* from southern Tibet and northwestern Yunnan, especially close to the individual from northwestern Yunnan (voucher No. ZXZ11027) (Fig. 2). Based on molecular and morphological evidence, we concluded that “*teng zhu*” should be identified as *Neomicrocalamus prainii*.

Li (1994) inferred that an ecogeographical diagonal line from northwestern Yunnan to southeastern Yunnan was caused by a northward and clockwise rotation of the Shan–Malay Plate (Burma–Malaya Geoblock) since the Miocene, and that northwestern and southeastern Yunnan were once at the same latitude both with a tropical environment. One of the biological effects of this plate movement was that some species were discontinuously distributed at both ends of the diagonal and some were concentrated on the southwestern side (Li et al. 1999, Zhu and Yan 2002). The disjunct distribution of *Neomicrocalamus prainii* in northwestern and southeastern Yunnan are concordant with this biogeographical line (Fig. 5). *Neomicrocalamus prainii* is distributed at both ends of the diagonal. The distribution pattern of *Neomicrocalamus prainii* may imply that this species has a tropical origin, and it provides another example for verifying the reality of the ecogeographical diagonal line in Yunnan.

### Melocalamus yunnanensis

This species was initially described as *Racemobambos yunnanensis* based on incomplete, poor specimens (Wen 1986). However, it is easily identified by observing the peculiar culm leaves. It was transferred to the genus *Neomicrocalamus* (Ohrnberger 1999), and subsequently subsumed into *Melocalamus* (Yi et al. 2007). Molecular studies also showed that it was not a member of *Racemobambos* or *Neomicrocalamus* and had a close relationship with *Melocalamus* (Yang et al. 2008). Main inflorescence features of *Melocalamus* include pseudospikelets 2-flowered, glumes 2, palea 2-keeled, lodicules 3, stamens 6, stigmas 2 or 3, plumose. *Melocalamus yunnanensis* as a member of the genus *Melocalamus* was confirmed by inflorescence characters reported in the current paper.

## Supplementary Material

XML Treatment for Melocalamus yunnanensis
